# Development and Validation of an Accurate Creatinine-Based Equation to Estimate Glomerular Filtration Rate for the Adult Indian Population: Design and Methods

**DOI:** 10.25259/IJN_221_2024

**Published:** 2024-09-18

**Authors:** Ashok Kumar Yadav, Arpita Ghosh, Vivek Kumar, Sreejith Parameswaran, Sitanshu Sekhar Kar, Jarnail Singh Thakur, Harbir Singh Kohli, Neil R Dalton, Tazeen H Jafar, Andrew S Levey, Vivekanand Jha

**Affiliations:** 1 Department of Experimental Medicine and Biotechnology, Postgraduate Institute of Medical Education and Research, Chandigarh, India; 2 George Institute for Global Health India, New Delhi, India; 3 Manipal Academy of Higher Education, Manipal, India; 4 University of New South Wales, Sydney, New South Wales, Australia; 5 Department of Nephrology, Postgraduate Institute of Medical Education and Research, Chandigarh, India; 6 Departments of Nephrology, Jawaharlal Institute of Postgraduate Medical Education & Research, Pondicherry, India; 7 Department of Preventive & Social Medicine, Jawaharlal Institute of Postgraduate Medical Education & Research, Pondicherry, India; 8 Department of Community Medicine, School of Public Health, Postgraduate Institute of Medical Education & Research, Chandigarh, India; 9 WellChild Laboratory, Evelina London Children’s Hospital, Guy’s and St Thomas NHS Foundation Trust, UK; 10 Program in Health Services & Systems Research, Duke-NUS Medical School, Singapore; 11 Division of Nephrology, Tufts Medical Center, Boston, Massachusetts, USA; 12 School of Public Health, Imperial College, London, UK

**Keywords:** Chronic kidney disease, eGFR, CKD-EPI, Lohexol

## Abstract

**Background:**

Existing creatinine-based equations to estimate glomerular filtration rate (GFR), developed primarily in populations of European and African American ancestry, do not accurately reflect the GFR in the Indian population due to differences in body composition, diet, and other factors. This manuscript describes the rationale and methodology for developing a creatinine-based equation for more accurate GFR estimation in Indian subjects.

**Materials and Methods:**

This cross-sectional study will be conducted in India’s two geographically and demographically diverse locations: Chandigarh (north) and Puducherry (south). Participants will include a representative sample from the general population and subjects with chronic kidney disease (CKD), with the latter being recruited from outpatient clinics. A total of 1558 subjects will be enrolled in the discovery and cross-validation cohort and 620 subjects in the external validation cohort. The reference standard for measured GFR (mGFR) will be the plasma clearance of iohexol. Stepwise multiple regression on log-transformed data will determine a set of variables that jointly predict mGFR and identify factors influencing mGFR and estimated (eGFR) in the study population. This study will also explore the performance of mGFR by iohexol measurement from dried blood spots against mGFR from plasma clearance of iohexol.

**Conclusion:**

Developing a more reliable and accurate creatinine-based GFR estimating equation will improve CKD diagnosis, classification, and management. The findings will have substantial implications for CKD research in India and other regions with similar populations.

## Introduction

Accurate estimates of the incidence and prevalence of chronic kidney disease (CKD) are unavailable for India, which is home to one-sixth of the global population. Small studies have shown an 8–10% population prevalence.^1–3^ According to the Global Burden of Disease (GBD) study, 15 million people had CKD in India in 2017, with about 230,000 attributable deaths.^4^ With a projected rise in the number of subjects with diabetes and hypertension, and the impact of emerging threats like environmental change, the number of people living with CKD is set to grow. Studies have shown a higher (18–22%) prevalence in geographies called CKD hot spots.^5,6^ There have been calls for population-wide screening for CKD.^7^ This requires the availability of tests that can establish abnormalities in kidney function accurately and reproducibly. Demonstrating a persistent decrease in the glomerular filtration rate (GFR) is a critical component of the CKD definition and classification.^8^ Determination of GFR is important for predicting the risk of kidney disease progression, listing for kidney transplantation, screening prospective kidney donors, determining drug dosing, and in studies where kidney function is the primary outcome.

### Measurement and estimation of GFR

Clearance measurements to ascertain measured GFR (mGFR) are necessary for research but impractical in clinical practice as they are costly, laborious, difficult to standardize, and require resources beyond those available in clinical laboratories. The reference standard method for mGFR is the urinary clearance of inulin. The complexity of the procedure, high cost, and unavailability of inulin have shifted the focus to alternative clearance procedures and exogenous markers. Most of these markers such as iothalamate, technetium 99m diethylenetriamine pentaacetic acid (99mTc-DTPA), and chromium 51-ethylenediamine tetraacetic acid (51Cr-EDTA) accurately measure the GFR; however, these markers contain radioisotopes, which makes its use problematic due to logistics such as storage and disposal of radioactive material, risk of exposure to study subjects, especially healthy young individuals, and high cost. In contrast, iohexol is a nonionic contrast agent, characterized by low protein binding, low extra-renal elimination, and no kidney tubular secretion or reabsorption which make iohexol a convenient alternative for GFR determination.^9^ Plasma clearance of iohexol, close to the reference standard method, is currently used for GFR measurement.

An ideal endogenous marker to ascertain estimated GFR (eGFR) should exhibit the same properties as an ideal exogenous marker, with a constant production rate and a simple, standardized, cost-effective measurement approach.^10^ Serum creatinine fulfils many of these criteria and is the most commonly used endogenous marker for eGFR. Measurements have been standardized using international reference standards and standardized methods. Kidney Disease: Improving Global Outcomes (KDIGO) guidelines recommends using a creatinine-based eGFR to assess kidney function.^8^ Cystatin C,^11,12^ a low molecular weight protein, is an alternative endogenous filtration marker with an international reference standard, but its measurement is more expensive than creatinine. Other endogenous filtration markers include low molecular weight proteins, beta-2 microglobulin (B2M), beta-trace protein (BTP),^13–16^ and other metabolites,^17–19^ but methods to measure these endogenous markers are not standardized.

### Accuracy of creatinine-based eGFR equations

The currently used creatinine-based eGFR equations were developed primarily amongst people of European and North American populations of European and African American ancestry. The predictive ability of the equations was not uniform, and a correction factor was needed for the African American populations. While the proportion of eGFR within 30% of mGFR (P_30_) was approximately 80% in validation populations, it was substantially less when tested in populations around the world: P_30_ using the MDRD equation was 54% (Japan), 68% (Thailand), 50% (Korea) and 58% (China); and 76% (Pakistan) and 62% (Thailand) using the CKD-EPI 2009 equation.^20–24^ The lesser accuracy was thought to be related to the influence of non-GFR determinants and stimulated the development of new equations or correction factors to the original equation.^8,21^ The 2024 KDIGO guideline update recommends using a validated GFR estimating equation to derive GFR from serum filtration markers rather than relying on the serum filtration markers alone. It suggests using the same equation within geographical regions as defined locally (e.g., continent, country, region).^25^

The Indian population differs from the Western population in being predominantly vegetarian, comparatively shorter, having less muscle mass, and a greater prevalence of central (abdominal) obesity.^26^ These factors likely alter the relationship between serum creatinine and GFR. Few studies have shown that the average mGFR in healthy Indian subjects is lower when compared to other ethnicities or racial groups.^27–30^ Comparison of eGFR with mGFR based on plasma clearance of DTPA in healthy kidney donors in India showed poor correlation.^27,31^ We showed that the CKD-EPI 2009 equation performed poorly against mGFR using urinary inulin clearance (mean bias; −24.92 ml/min/1.73 m^2^, accuracy; P_30_: 22.3%). Another notable finding was the relatively low average GFR in healthy study participants (79 ml/min/1.73 m^2^) compared to 100–130 ml/min/1.73 m^2^ described in the Western populations.^32^ Further, the low GFR in Indian subjects may account for low protein intake, as preserved renal functional reserve have been shown with acute rise in GFR with protein loading.^30^ This finding needs to be confirmed in larger population-based studies as it has implications for defining the threshold GFR for diagnosis of kidney disease in the Indian population.

The current GFR measurement methods involve bringing the subject to a specialized facility, which restrict its use to hospital or research centers. Attempts are being made to standardize dried blood spot (DBS) sampling, a minimally invasive technique that involves blotting and drying blood samples on filter paper. The spots are stable at room temperature for a long duration and can be shipped to a laboratory without stringent transport requirements.^33,34^ The technique is easy to perform, less painful, requires minimal blood volume, and can be done in outpatient clinics and community settings.

Considering the need to determine the level of mGFR in the Indian population and to develop more accurate and simpler tools to estimate GFR that would help in the diagnosis, classification, and risk stratification of subjects with CKD, this study aims to fill the information void in the study of the epidemiology of CKD in India.

### Objectives

The primary objective is to derive and validate a creatinine-based eGFR equation and/or a correction factor for the existing CKD-EPI equation, with multiple sample plasma clearance of iohexol as the reference standard.

The secondary objectives are to ascertain the range of GFR in subjects drawn from the community, assess the performance of GFR calculated from dried blood spot iohexol clearance against multiple sample plasma iohexol clearance, and ascertain explanatory factors associated with reference standard mGFR and eGFR.

## Materials and Methods

This study has a cross-sectional design. A sample drawn from the general population and subjects with CKD will be used for derivation and cross-validation of the eGFR equation. A second set of samples from the general population and subjects with CKD will be used for external validation. Ethical approval was obtained.

This study will be conducted in Chandigarh (North India) and Puducherry (South India).

### Screening

For community-based sampling, adult (≥18 years) residents of selected sites in Chandigarh and Puducherry will constitute the target population. CKD subjects will be drawn from the outpatient clinics of the Postgraduate Institute of Medical Education and Research (PGIMER), Chandigarh, and Jawaharlal Institute of Postgraduate Medical Education and Research (JIPMER), Puducherry. Details of the inclusion and exclusion criteria are mentioned in Table 1.

### Justification for choice of study population

While the uncertainty around GFR estimation is greater at upper GFR ranges, developing an accurate equation requires including subjects at the lower end of the range of GFR. For clinical application, it is important to ensure that the population in which the equation is to be developed will represent the population in which it will be applied. Therefore, this study will recruit representative subjects from the entire range of GFR. The greatest public health benefit, however, is likely to come from the appropriate identification of the early stages of kidney disease.

### Selection of subjects for screening

This study will employ multistage sampling. Chandigarh has 55 sectors (urban) and 11 villages (rural). Puducherry has 90 wards (urban) and 62 villages (rural). Sectors, wards, and villages will constitute the primary sampling units (PSUs). Twenty sectors and four villages in Chandigarh and 14 wards and 12 villages in Puducherry will be selected by stratified random sampling (SRS). Next, 15 households from each selected PSU will be selected by SRS, giving a total of 750 households. Of all the household members between the ages of 18 and 64, one individual will be selected from each household by the “KISH method.”^35^ If the household has at least one 65-plus individual, one of them will be chosen randomly.

Subjects with CKD will be enrolled in the clinics of the two hospitals. In all, 750 subjects with eGFR ≥15–90 ml/min/1.73m^2^ (630 between 18 and 64 years and 120 >65 years) will be enrolled for development and cross-validation and 500 for external validation.

### Study procedures

The study staff will visit selected households and explain the study procedure to eligible and willing subjects. After obtaining written consent, plasma clearance of iohexol will be scheduled at a central facility (PGIMER, Chandigarh, or JIPMER, Puducherry). Participants will be instructed on collecting 24-hour urine samples a day before the procedure and asked to report in a fasting state.

Dietary history, demographic details, and anthropometric data will be recorded on the test day. Body composition will be analyzed by a multifrequency bioimpedance analyzer (BC-420MA, TANITA Corporation, UK) validated for medical research.

### Procedure, measurement of Iohexol, and calculation of mGFR

After pre-procedure oral hydration with 500 ml water, venous blood sample will be drawn for serum creatinine estimation. Plasma and serum will also be aliquoted and stored at −80°C.

**Fig. 1:**
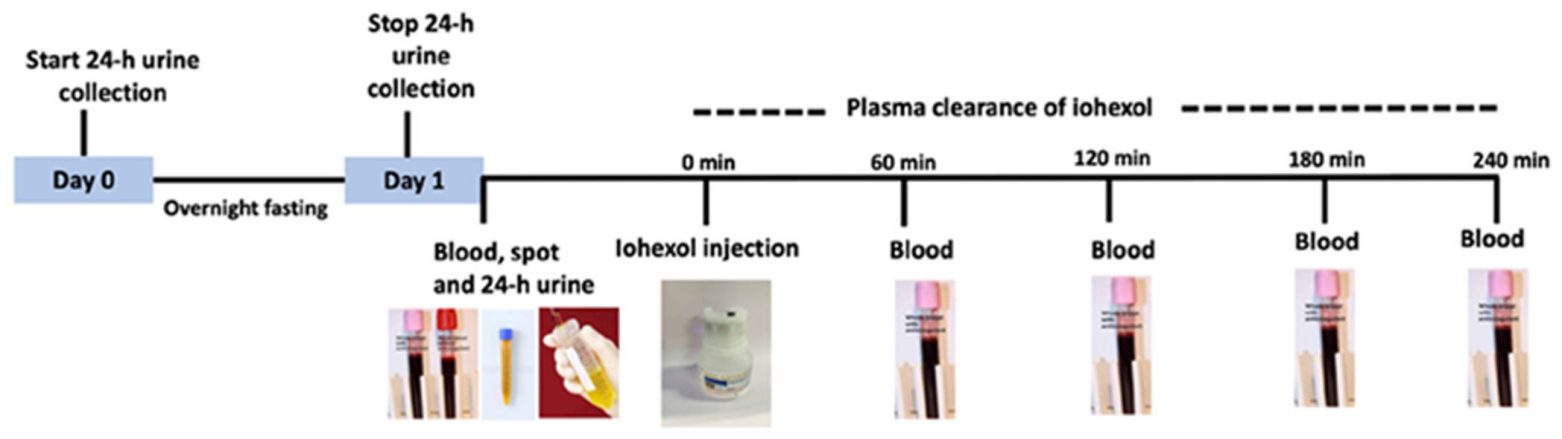
Graphical representation of methodology for sampling procedure for measurement of GFR using iohexol clearance. GFR: Glomerular filtration rate.

Participants will be given 300 mg/ml of OMNIPAQUE (iohexol) by slow intravenous injection over two minutes, followed by flushing with 1 ml saline. OMNIPAQUE dose will be 5 ml and 3 ml in subjects with body weight ≥50kg and <50 kg, respectively. The syringe will be weighed before and after injection. The amount of iohexol will be calculated from the difference in syringe weight multiplied by the concentration of iohexol divided by its density at room temperature.^36^ Plasma samples will be taken from the contralateral arm peripheral vein pre-procedure and at 60, 120, 180, and 240 minutes [Figure 1] and be stored at −80°C. Finger prick samples will be collected at the same time points on the Whatman 903 filter paper. Samples will be collected to fill the entire circle and soak both sides of the paper. A 6.3 mm punch of blood spot will be used for analysis.

Iohexol will be measured in plasma and DBS as described.^37,34^ Iohexol will be extracted using an aqueous/dichloromethane extraction process. The concentration of the aqueous eluate will be determined using High-Performance Liquid Chromatography-Ultraviolet (HPLC-UV) detection at 254 nm at 45°C. The percentage recovery of iohexol will be determined by the addition of a known concentration of iopentol before the extraction. This will act as an internal standard. For DBS, iohexol concentrations will be calculated assuming a fixed blood volume (6.3 mm punch ≈11.2 µL of blood). The equivalent value for serum will be calculated using the formula: (iohexol)/(1-hematocrit). The final absolute concentration of iohexol will be calculated using the height of the peak of the second isomer of iohexol and the peak of internal control and standards. The iohexol methodology will be validated using an external quality control program (Equalis AB, Sweden).

To calculate the GFRs, the one-pool clearance model^38^ will be used, and GFR will be corrected for body surface area.^39^

Serum and urine creatinine will be measured using the modified Jaffe’s kinetic method, which is traceable to the reference isotope dilution mass spectrometry (IDMS) standard method. Urine creatinine will be measured to ensure the accuracy of the 24-hour urine sample collection. Serum cystatin C will be measured using nephelometric immunoassay with calibration traceable to the internationally certified reference material ERM-DA471/IFCC. Dietary protein intake will be calculated from the 24-hour urine urea nitrogen appearance (UNA) rate.^28,40,41^

### Statistical considerations

Continuous data will be presented as mean ± standard deviation (SD) and median (interquartile range). Categorical data will be presented as counts and percentages. To compare distributions of factors in two independent samples, Chi-square, t-test, and Mann-Whitney U test will be used. The sensitivity and specificity of GFR estimates will be determined to identify mGFR <60 mL/min/1.73 m^2^. Receiver operating characteristic curves will be constructed, and the area under the curve (AUC) will be calculated for GFR estimate identifying mGFR <60 mL/min per 1.73 m^2^. Bland-Altman plots will be used to examine the agreement between mGFR and eGFR estimates in the validation dataset.

### Derivation of eGFR equation

We will use stepwise multiple regression on log-transformed data to determine a set of variables that jointly predict mGFR. The prediction equation will be a multiplicative model. The regression coefficients of the model will refer to the change in geometric mean mGFR associated with changes in the independent variable. Estimated GFR will be expressed in ml/min/1.73 m^2^. Age, sex, weight, height, north or south Indian origin, and serum creatinine will be considered for inclusion in the regression model—first main effects and then interactions between variables. The forms of the continuous predictors will be explored using flexible approaches such as fractional polynomials and restricted cubic splines. As they are related inversely, mGFR and serum creatinine will be log-transformed. A P-value <0.01 will be used as the criterion for entry of a variable into the model. Other models that use additional variables will be considered: waist circumference, total body fat, muscle mass, serum albumin, fasting blood glucose, and serum urea nitrogen.

### Development of a correction factor for CKD-EPI Equation

A linear regression model will be created for log-transformed mGFR against log-transformed eGFR using CKD-EPI equations. Intercept and slopes that are statistically significant (P <0.05) will be back-transformed to exponential form and used as correction factors for CKD-EPI equation resulting in CKD-EPI India and referred to as CKD-EPI_Ind_.

### Metrics of equation performance

Bias, precision, root mean square error (RMSE) and accuracy (P_30_) with 95% confidence intervals of the new eGFR equation against mGFR will be calculated. Bias will be expressed as the median of the differences between mGFR and eGFR, and precision will be expressed as the interquartile range of the differences. The square root of the average squared difference between mGFR and eGFR will give the RMSE. A 95% limits of agreement will be calculated as mean bias ± 1.96 x SD. Accuracy will be expressed as RMSE of the difference between mGFR and eGFR and the percentage of subjects with eGFR within ±30% of mGFR (P_30_). Confidence intervals for estimates will be calculated using the bootstrap resampling method. The equation with the best metrics, that is, the combination of lowest bias, RMSE, and highest P_30_, will be accepted. Performance will be evaluated in subgroups defined by the variables described above.

The difference between the estimates of the new equation and the existing eGFR equations will be tested by examining the 95% CIs and paired t-test. It will be considered significant when the 95% CIs do not overlap or the p-value of the t-test is <0.05.

### Cross-validation of the developed equation

We will use 50 iterations of the tenfold cross-validation to assess the model performance of the newly developed prediction equation using the 1558 samples. The original sample will be randomly partitioned into ten equal subsamples. Of the ten subsamples, a single subsample will be used as the test set for validation and the remaining nine subsamples will be used as training data for developing the model using stepwise regression as described earlier—this will be done ten times such that each subsample is used as test data once. This whole process will be repeated 50 times. The estimates from the 50 runs (i.e., total 500 bootstrap samples) will be combined to produce a single estimate of model performance.

### External validation

The final generalizable equation will be tested in separate datasets other than the development dataset. These will be another general population cohort of 120 subjects split equally between the two sites and 500 subjects enrolled in the ICKD study^42^ at PGIMER and JIPMER. The performance of the new eGFR equation will be assessed by bias, precision, and accuracy against mGFR.

### DBS iohexol measurement

Bias, precision, and accuracy of mGFR by plasma iohexol clearance using DBS sampling will be assessed against venous blood plasma iohexol mGFR (reference method). The agreement between the two values will be assessed by the concordance correlation coefficient (CCC), total deviation index (TDI), and coverage probability (cp). Agreement between the reference and DBS methods will be evaluated using Bland-Altman plots.

### Factors associated with mGFR

We will examine the association between mGFR and age, sex, North or South Indian origin, dietary protein intake (as assessed by UNA), 24-hour urinary creatinine excretion, muscle mass, and total fat mass.

### Factors associated with non-GFR determinants of serum creatinine

We will examine the following factors associated with the non-GFR determinants of serum creatinine: age, sex, height, weight, body mass index, muscle mass (as assessed by bioimpedance), dietary protein intake, 24-hour urinary creatinine excretion, hypertension, diabetes, glucose, total cholesterol, serum sodium, potassium, bicarbonate, calcium, and phosphate. The relationship of serum creatinine with the predictor variables will be analyzed by regressing log-transformed creatinine on the predictor variables adjusting for log-transformed GFR and accounting for mGFR measurement error and the interaction between GFR and predictor variables.

### Sample size calculation

To develop a prediction equation to estimate GFR based on independent variables using multiple linear regression, a sample size of 252 achieves 90% power to detect an R^2^ of 0.10 attributed to 20 independent variables using an F-test with a significance level (alpha) of 0.05. To account for the clustered sampling design, we multiply this sample size by a design effect of 2, leading to a sample size of 504. With 20% dropout, we need 630 participants. We will use a similar sample size of n = 630 for subjects with CKD. The external validation set includes another 120 general population participants. We will, therefore, sample 750 households from 14 wards and 12 villages in Puducherry and 20 sectors and 4 villages in Chandigarh. We will sample another set of 500 subjects with CKD from PGIMER and JIPMER for the external validation cohort.

To ensure that the elderly population (65 years or more) is adequately represented in our sample, we will select 178 individuals with age >65 years from each sampled household that has at least one 65-plus individual. Similarly, 120 CKD subjects with age >65 will be enrolled. Thus, the total sample size for the study will include 928 participants from the general population and 1250 subjects with CKD.

A secondary objective is to estimate the mean mGFR in the general population. A sample of 387 participants will allow us to estimate the GFR mean to within ± 2 ml/min/1.73 m^2^ with 95% confidence when the SD is assumed to be 20 ml/min/1.73 m^2^.

### Data management

A specific database management system will be developed. Data protection would be implemented according to the Digital Information Security in Healthcare Act (DISHA) 2022 guidelines. Identifiable personal data will be held separately from the study database and the link files will be stored on a separate hard disk drive. All data will be stored in a password-protected computer with restricted access. Data will be curated in a relational database, labeled, formatted, and documented consistently over time and across samples.

## Discussion

This study will lead to an improved understanding of the GFR distribution and its determinants, the development of a new creatinine-based eGFR equation and/or a correction factor for the existing CKD-EPI equations, and factors associated with the error in eGFR for the adult Indian population. As described above, this will allow for more accurate diagnosis and classification of subjects with CKD and improve risk prediction for progression to more advanced kidney disease in Indian subjects. This information is also of value in the appropriate selection of living kidney donors. Including subjects from North and South India and the entire range of GFR will allow this study’s results to be broadly representative of the country. If validated successfully, DBS sampling will help perform GFR measurements in large-scale epidemiological studies.

CKD is one of the focus areas in the overall noncommunicable disease strategy of the Government of India.^43^ There have been calls from the Indian Nephrology community to set up CKD detection and treatment programs throughout the country, given the rising prevalence of CKD and its adverse consequences.^7^ Community-level screening has been demanded in areas showing a particularly high burden of CKD of undetermined etiology that develops without known risk factors. This study will be the first to document the distribution of GFR in the Indian population. This knowledge is relevant for interpreting GFR data and determining whether people with GFR values considered below normal range as determined by the Western standards are at high risk of adverse outcomes and should receive interventions to slow down the progression of CKD or whether these low values are physiological. This will require following these individuals over a long period.

The availability of an accurate GFR estimating equation is crucial to the success of this strategy. Examples from countries such as Japan, China, and Thailand show that the estimates of the prevalence of CKD had to be revised by using a region-specific GFR equation. To date, no attempts have been made to validate or develop eGFR equations based on current standards in the Indian population.^44^

This study has limitations. Although it includes participants from one Northern and one Southern state, it does not represent the Indian population in its entirety. This study is cross-sectional in nature; so while it will allow us to determine the range of GFR in the population studied, it will not be able to determine the physiological significance of any low GFR values encountered in the general population in the absence of any other abnormality, which will require following up the population to determine the influence on outcomes.

## Conclusion

This study will develop an accurate eGFR estimating equation and/or modification in the existing eGFR equation to estimate GFR in the adult Indian population accurately. This study will help us to determine the range of GFR as well as factors associated with mGFR and factors associated with errors in eGFR in the adult Indian population. The findings could have substantial implications for CKD research in India and other regions with similar population characteristics.

## Figures and Tables

**Table 1:** Inclusion and exclusion criteria for enrollment of general population from community and CKD subjects from hospital

General population	CKD subjects
Inclusion criteria	Inclusion criteria
Age >18 years	Age >18 years
Stable clinical state for the last three months as judged by the investigator	eGFR (CKD-EPI creatinine equation 2009) range from ≤60 ml/min/1.7 3m^2^ to ≥ 15 ml/min/1.73 m^2^ and >60 to ≤ 90 ml/min/1.73 m^2^ with proteinuria (urine protein to creatinine ratio >500 mg/g or albumin to creatinine ratio >300 mg/g)
No history of any ongoing chronic illness	Stable clinical state for the last three months as judged by the investigator
Able to consent	Venous sampling possible for both upper limbs Able to consent
Exclusion criteria	Exclusion criteria
History of kidney stone disease or urinary tract disease Voiding difficulty or incontinence Amputation in any limb Hospitalization in the last three months Poor functional status as judged by investigator.	Dialysis dependency or having received dialysis in the preceding three months Active malignancy or treatment for malignancy in the last two years Voiding dysfunction or problems Urinary incontinence
Bedridden subjects	Pregnancy
Pregnancy or lactation in case of females	Allergy to iohexol or iodinated contrast media
History of allergy to iodinated contrast media	Amputation of any limb Decompensated chronic liver disease or heart failure Currently receiving antibiotic therapy for systemic infection Positive HIV antibody or positive Hepatitis surface antigen RBC transfusion within eight weeks before enrollment Androgen therapy within eight weeks before enrollment Any chronic unstable medical condition History of drug abuse Sleep apnea Bone and joint abnormalities that were preclude to exercise testing

CKD: chronic kidney disease, eGFR: estimated glomerular filtration rate, CKD-EPI: Chronic kidney Disease Epidemiology Collaboration, RBC: Red blood cells
